# 1-(2-Furo­yl)-3-(2-meth­oxy-4-nitro­phen­yl)thio­urea

**DOI:** 10.1107/S1600536813002894

**Published:** 2013-02-02

**Authors:** Seema Pratap, Durga P. Singh, Sushil K. Gupta, Sema Öztürk Yildirim, Ray J. Butcher

**Affiliations:** a Department of Chemistry, M. M. V., Banaras Hindu University, Varanasi 221 005, India; b School of Studies in Chemistry, Jiwaji University, Gwalior 474 011, India; c Department of Physics, Faculty of Sciences, Erciyes University, 38039 Kayseri, Turkey; d Department of Chemistry, Howard University, 525 College Street NW, Washington, DC 20059, USA

## Abstract

The asymmetric unit of the title compound, C_13_H_11_N_3_O_5_S, contains two independent mol­ecules, which are linked by a pair of inter­molecular N—H⋯S hydrogen bonds, forming an *R*_2_^2^(8) ring motif. The central thio­urea core forms dihedral angles of 3.02 (12) and 14.00 (10)° with the essentially planar furoyl groups [maximum deviations = 0.030 (2) and 0.057 (2) Å] in the two mol­ecules and dihedral angles of 2.43 (13) and 8.03 (12)° with the benzene rings. The dihedral angles between the furoyl and benzene rings in the two mol­ecules are 3.97 (10) and 5.98 (9)°. The *trans–cis* geometry of the thio­urea group is stabilized by three intra­molecular N—H⋯O hydrogen bonds involving carbonyl and meth­oxy O atoms with the H atom of the *cis*-thio­amide group and between furan O atom and the other thio­amide H atom. There is also a weak intra­molecular C—H⋯S inter­action in each mol­ecule.

## Related literature

For background to anion receptors, see: Doyle & Jacobsen (2007 ▶); Gale *et al.* (2008 ▶); Svetlana (2007 ▶). For aroyl thio­ureas as ionophores, see: Wilson *et al.* (2010 ▶); Pérez *et al.* (2008 ▶) and as catalysts, see: Yang *et al.* (2004 ▶); Dai *et al.* (2004 ▶). For related structures, see: Koch (2001 ▶); Pérez *et al.* (2008 ▶); Singh *et al.* (2012*a* ▶,*b* ▶,*c* ▶). For standard bond lengths, see: Allen *et al.* (1987 ▶). For hydrogen-bond motifs, see: Bernstein *et al.* (1995 ▶).
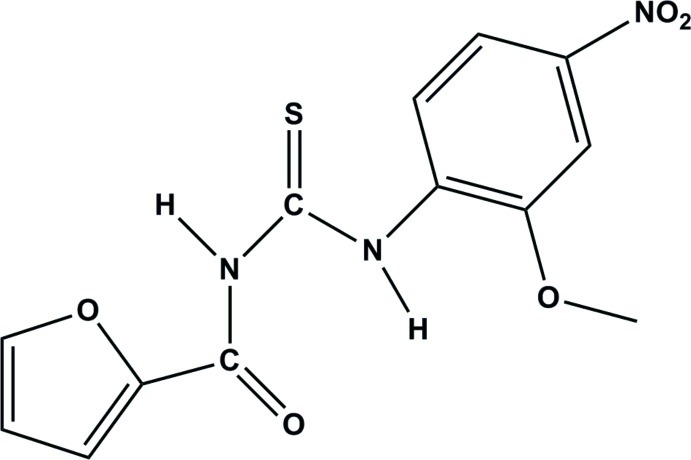


## Experimental

### 

#### Crystal data

C_13_H_11_N_3_O_5_S
*M* *_r_* = 321.31
Triclinic, 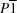

*a* = 7.9474 (6) Å
*b* = 13.0122 (10) Å
*c* = 13.4215 (11) Å
α = 87.734 (6)°
β = 77.014 (7)°
γ = 86.945 (7)°
*V* = 1350.00 (18) Å^3^
*Z* = 4
Cu *K*α radiation
μ = 2.43 mm^−1^
*T* = 123 K
0.69 × 0.21 × 0.04 mm

#### Data collection

Agilent Xcalibur (Ruby, Gemini) diffractometer
Absorption correction: analytical [*CrysAlis PRO* (Agilent, 2012 ▶), based on expressions derived by Clark & Reid (1995 ▶)] *T*_min_ = 0.441, *T*_max_ = 0.909
9239 measured reflections
5400 independent reflections
4064 reflections with *I* > 2σ(*I*)
*R*_int_ = 0.042

#### Refinement

*R*[*F* ^2^ > 2σ(*F* ^2^)] = 0.046
*wR*(*F* ^2^) = 0.134
*S* = 1.03
5400 reflections
399 parameters
H-atom parameters constrained
Δρ_max_ = 0.36 e Å^−3^
Δρ_min_ = −0.34 e Å^−3^

### 

Data collection: *CrysAlis PRO* (Agilent, 2012 ▶); cell refinement: *CrysAlis PRO*; data reduction: *CrysAlis PRO*; program(s) used to solve structure: *SHELXS97* (Sheldrick, 2008 ▶); program(s) used to refine structure: *SHELXL97* (Sheldrick, 2008 ▶); molecular graphics: *SHELXTL* (Sheldrick, 2008 ▶); software used to prepare material for publication: *SHELXTL*.

## Supplementary Material

Crystal structure: contains datablock(s) global, I. DOI: 10.1107/S1600536813002894/lh5568sup1.cif

Structure factors: contains datablock(s) I. DOI: 10.1107/S1600536813002894/lh5568Isup2.hkl

Supplementary material file. DOI: 10.1107/S1600536813002894/lh5568Isup3.cml

Additional supplementary materials:  crystallographic information; 3D view; checkCIF report

## Figures and Tables

**Table 1:** Hydrogen-bond geometry (Å, °)

*D*—H⋯*A*	*D*—H	H⋯*A*	*D*⋯*A*	*D*—H⋯*A*
N1*A*—H1*AA*⋯O1*A*	0.88	2.24	2.684 (2)	111
N2*A*—H2*AA*⋯O2*A*	0.88	1.91	2.654 (2)	142
N2*A*—H2*AA*⋯O3*A*	0.88	2.09	2.552 (2)	112
N1*B*—H1*BA*⋯O1*B*	0.88	2.25	2.683 (2)	111
N2*B*—H2*BA*⋯O2*B*	0.88	1.92	2.653 (2)	140
N2*B*—H2*BA*⋯O3*B*	0.88	2.11	2.554 (2)	111
C8*A*—H8*AA*⋯S1*A*	0.95	2.52	3.198 (2)	129
C8*B*—H8*BA*⋯S1*B*	0.95	2.52	3.189 (2)	128

**Fig. 1.**
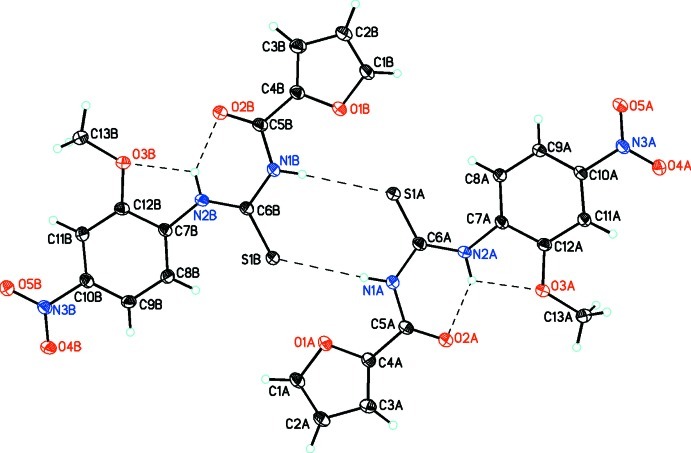
Molecular structure of the title compound showing 30% probability displacement ellipsoids. Dashed lines indicate an intramolecular N—H···O and intermolecular N—H···S hydrogen bonds.

## supplementary crystallographic information

### Comment

There is a growing interest in the synthesis of new substituted thiourea derivatives owing to their diverse applicability in the pharmaceutical industry, material science and analytical chemistry. The hydrogen-bonding ability of the thiourea moiety has extensively been used in construction of anion receptors (Doyle & Jacobsen, 2007; Gale *et al.*, 2008; Svetlana 2007). Further, aroyl thioureas have been successfully used in environmental control, as ionophores in ion-selective electrodes (Wilson *et al.*,2010; Pérez *et al.*, 2008). Recently, these compounds have been employed successfully as catalysts in the palladium-catalyzed Suzuki and Heck reactions (Yang *et al.*, 2004; Dai *et al.*, 2004). In view of the above and in continuation of our work on thiourea derivatives (Singh *et al.*, 2012*a*,*b*,*c*), the crystal structure of 1-(2-furoyl)-3-(2-methoxy- 4-nitrophenyl)thiourea has been determined (Fig.1).

The asymmetric unit of the structure contains two molecules, which are linked by a pair of intermolecular N—H···S hydrogen bonds forming an *R*^2^_2_(8) motif. The main bond lengths are within the ranges obtained for similar compounds (Koch 2001; Pérez *et al.*, 2008, Singh *et al.* 2012*c*). The C6A—S1A [1.667 (2) Å], C6B—S1B [1.665 (2) Å] and C5A—O2A [1.226 (3) Å], C5B—O2B [1.224 (3) Å] bonds show typical double-bond character. However, the C—N bond lengths, C5A—N1A [1.390 (3) Å], C6A—N1A [1.391 (3) Å], C6A—N2A [1.349 (3) Å], C7A—N2A [1.405 (3) Å] and C5B—N1B [1.389 (3) Å], C6B—N1B [1.397 (3) Å], C6B—N2B [1.348 (3) Å], C7B—N2B [1.406 (3) Å] are shorter than the normal C—N single-bond length of about 1.48 Å (Allen *et al.*, 1987). These results can be explained by the existence of resonance in this part of the molecule. The essentially planar furoyl groups (C1A-C5A/O1A/O2A with maxixum deviation of 0.030 (2)Å for O2A) and (C1B-C5B/O1B/O2B with maximum deviation of 0.057 (2)Å for O2B) groups are inclined at angles of 3.02 (12)° and 14.00 (10)° with respect to the plane formed by the thiourea group (N1/N3/C6/S1), whereas the benzene (C7A-C12A and C7B-C12B) rings are inclined at angles of 2.43 (13)° and 8.03 (12)° with the thiourea plane, respectively. The dihedral angles in two independent molecules between the furoyl groups and benzene rings are 3.97 (10)° and 5.98 (9)°, respectively. The *trans-cis* geometry in the thiourea moiety is stabilized by three intramolecular N—H···O hydrogen bonds involving carbonyl (O2A/O2B) and methoxy (O3A/O3B) O atoms with the H atom of the *cis*-thioamide group and between furan (O1A/O1B) O atom and the other thioamide H atom (Table 1). In addition, an intramolecular C—H···S interaction is also observed in each molecule (Table 1).

### Experimental

A solution of 2-furoyl chloride (0.01 mol) in anhydrous acetone (80 ml) was added drop wise to a suspension of ammonium thiocyanate (0.01 mol) in anhydrous acetone (50 ml) and the reaction mixture was heated to reflux for 50 minutes. After cooling to room temperature, a solution of 2-methoxy-4-nitroaniline (0.01 mol) in dry acetone (25 ml) was added slowly and the resulting mixture refluxed for 2 h. The reaction mixture was poured into five times its volume of cold water, upon which the thiourea precipitated. The resulting solid product was crystallized from dimethyl sulphoxide yielding light yellow X-ray quality single crystals. Yield: 82%; M.P.: 451–453 K. Anal. Calc. for C_13_H_11_N_3_O_5_S (%): C, 48.59; H, 3.45; N, 13.07. Found: C, 48.40; H, 3.48; N, 12.96.

### Refinement

All H atoms were placed in calculated positions and refined using a riding-model approximation with C—H = 0.95-0.98 Å, N—H = 0.88 Å and *U*_iso_(H) = 1.2 *U*_eq_(C,*N*) or 1.5 *U*_eq_(C_methyl_).

### Crystal data

**Table:**

C_13_H_11_N_3_O_5_S	*Z* = 4
*M**_r_* = 321.31	*F*(000) = 664
Triclinic, *P*1	*D*_x_ = 1.581 Mg m^−^^3^
Hall symbol: -P 1	Cu *K*α radiation, λ = 1.54184 Å
*a* = 7.9474 (6) Å	Cell parameters from 2786 reflections
*b* = 13.0122 (10) Å	θ = 3.4–75.6°
*c* = 13.4215 (11) Å	µ = 2.43 mm^−^^1^
α = 87.734 (6)°	*T* = 123 K
β = 77.014 (7)°	Long plate, colorless
γ = 86.945 (7)°	0.69 × 0.21 × 0.04 mm
*V* = 1350.00 (18) Å^3^	

### Data collection

**Table:**

Agilent Xcalibur (Ruby, Gemini) diffractometer	5400 independent reflections
Radiation source: Enhance (Cu) X-ray Source	4064 reflections with *I* > 2σ(*I*)
Graphite monochromator	*R*_int_ = 0.042
Detector resolution: 10.5081 pixels mm^-1^	θ_max_ = 75.8°, θ_min_ = 3.4°
ω scans	*h* = −6→9
Absorption correction: analytical [*CrysAlis PRO* (Agilent, 2012), based on expressions derived by Clark & Reid (1995)]	*k* = −15→16
*T*_min_ = 0.441, *T*_max_ = 0.909	*l* = −15→16
9239 measured reflections	

### Refinement

**Table:**

Refinement on *F*^2^	Primary atom site location: structure-invariant direct methods
Least-squares matrix: full	Secondary atom site location: difference Fourier map
*R*[*F*^2^ > 2σ(*F*^2^)] = 0.046	Hydrogen site location: inferred from neighbouring sites
*wR*(*F*^2^) = 0.134	H-atom parameters constrained
*S* = 1.03	*w* = 1/[σ^2^(*F*_o_^2^) + (0.0713*P*)^2^] where *P* = (*F*_o_^2^ + 2*F*_c_^2^)/3
5400 reflections	(Δ/σ)_max_ < 0.001
399 parameters	Δρ_max_ = 0.36 e Å^−^^3^
0 restraints	Δρ_min_ = −0.34 e Å^−^^3^

### Special details

**Table:**

Geometry. All e.s.d.'s (except the e.s.d. in the dihedral angle between two l.s. planes) are estimated using the full covariance matrix. The cell e.s.d.'s are taken into account individually in the estimation of e.s.d.'s in distances, angles and torsion angles; correlations between e.s.d.'s in cell parameters are only used when they are defined by crystal symmetry. An approximate (isotropic) treatment of cell e.s.d.'s is used for estimating e.s.d.'s involving l.s. planes.
Refinement. Refinement of *F*^2^ against ALL reflections. The weighted *R*-factor *wR* and goodness of fit *S* are based on *F*^2^, conventional *R*-factors *R* are based on *F*, with *F* set to zero for negative *F*^2^. The threshold expression of *F*^2^ > σ(*F*^2^) is used only for calculating *R*-factors(gt) *etc*. and is not relevant to the choice of reflections for refinement. *R*-factors based on *F*^2^ are statistically about twice as large as those based on *F*, and *R*- factors based on ALL data will be even larger.

### Fractional atomic coordinates and isotropic or equivalent isotropic displacement parameters (Å^2^)

**Table:**

	*x*	*y*	*z*	*U*_iso_*/*U*_eq_	
S1A	0.36097 (8)	0.11036 (4)	0.27285 (4)	0.03539 (16)	
S1B	0.59249 (8)	0.38403 (4)	0.24534 (4)	0.03632 (16)	
O1A	0.5944 (2)	0.35328 (11)	0.46572 (13)	0.0332 (3)	
O2A	0.3761 (2)	0.14473 (12)	0.60923 (13)	0.0352 (4)	
O3A	0.1883 (2)	−0.06917 (12)	0.63465 (12)	0.0338 (3)	
O4A	−0.1054 (3)	−0.37579 (13)	0.55069 (14)	0.0421 (4)	
O5A	−0.0861 (3)	−0.36147 (13)	0.38735 (14)	0.0407 (4)	
O1B	0.3641 (2)	0.14072 (11)	0.04830 (12)	0.0324 (3)	
O2B	0.6346 (2)	0.32954 (12)	−0.09340 (13)	0.0357 (4)	
O3B	0.8303 (2)	0.54209 (12)	−0.11942 (12)	0.0347 (4)	
O4B	1.0361 (2)	0.85635 (13)	0.13539 (13)	0.0387 (4)	
O5B	1.1180 (2)	0.84800 (13)	−0.02921 (13)	0.0398 (4)	
N1A	0.4231 (2)	0.18859 (14)	0.43709 (14)	0.0298 (4)	
H1AA	0.4660	0.2370	0.3924	0.036*	
N2A	0.2838 (2)	0.03587 (13)	0.46879 (14)	0.0283 (4)	
H2AA	0.2932	0.0474	0.5314	0.034*	
N3A	−0.0598 (2)	−0.33027 (13)	0.46753 (15)	0.0304 (4)	
N1B	0.5391 (2)	0.30242 (13)	0.08037 (14)	0.0299 (4)	
H1BA	0.4777	0.2607	0.1260	0.036*	
N2B	0.6826 (2)	0.45407 (13)	0.04855 (14)	0.0285 (4)	
H2BA	0.6776	0.4411	−0.0146	0.034*	
N3B	1.0386 (2)	0.81488 (14)	0.05402 (15)	0.0308 (4)	
C1A	0.6737 (3)	0.43079 (16)	0.49908 (19)	0.0344 (5)	
H1AB	0.7340	0.4830	0.4563	0.041*	
C2A	0.6548 (3)	0.42318 (17)	0.6015 (2)	0.0357 (5)	
H2AB	0.6978	0.4684	0.6428	0.043*	
C3A	0.5584 (3)	0.33487 (18)	0.63570 (19)	0.0344 (5)	
H3AA	0.5245	0.3090	0.7041	0.041*	
C4A	0.5245 (3)	0.29491 (16)	0.55114 (17)	0.0297 (4)	
C5A	0.4348 (3)	0.20312 (16)	0.53730 (17)	0.0288 (4)	
C6A	0.3526 (3)	0.10763 (16)	0.39819 (17)	0.0285 (4)	
C7A	0.1995 (3)	−0.05422 (15)	0.45914 (17)	0.0269 (4)	
C8A	0.1628 (3)	−0.09002 (16)	0.37023 (16)	0.0295 (4)	
H8AA	0.1967	−0.0521	0.3076	0.035*	
C9A	0.0774 (3)	−0.18036 (16)	0.37248 (17)	0.0302 (4)	
H9AA	0.0520	−0.2049	0.3120	0.036*	
C10A	0.0297 (3)	−0.23418 (15)	0.46458 (17)	0.0276 (4)	
C11A	0.0633 (3)	−0.20163 (16)	0.55529 (17)	0.0288 (4)	
H11A	0.0289	−0.2404	0.6174	0.035*	
C12A	0.1482 (3)	−0.11100 (16)	0.55243 (16)	0.0274 (4)	
C13A	0.1296 (3)	−0.1174 (2)	0.73323 (18)	0.0393 (5)	
H13A	0.1554	−0.0746	0.7859	0.059*	
H13B	0.1887	−0.1853	0.7353	0.059*	
H13C	0.0046	−0.1254	0.7460	0.059*	
C1B	0.2969 (3)	0.05868 (16)	0.01333 (19)	0.0340 (5)	
H1BB	0.2204	0.0125	0.0550	0.041*	
C2B	0.3542 (3)	0.05241 (17)	−0.0883 (2)	0.0361 (5)	
H2BB	0.3257	0.0021	−0.1304	0.043*	
C3B	0.4655 (3)	0.13491 (18)	−0.12144 (19)	0.0358 (5)	
H3BA	0.5265	0.1504	−0.1893	0.043*	
C4B	0.4665 (3)	0.18683 (16)	−0.03598 (18)	0.0303 (4)	
C5B	0.5544 (3)	0.27857 (16)	−0.02133 (17)	0.0298 (4)	
C6B	0.6086 (3)	0.38421 (15)	0.11951 (17)	0.0281 (4)	
C7B	0.7665 (3)	0.54421 (15)	0.05886 (17)	0.0266 (4)	
C8B	0.7740 (3)	0.58953 (16)	0.15028 (17)	0.0311 (4)	
H8BA	0.7187	0.5587	0.2137	0.037*	
C9B	0.8613 (3)	0.67881 (16)	0.14927 (17)	0.0313 (4)	
H9BA	0.8652	0.7101	0.2114	0.038*	
C10B	0.9425 (3)	0.72156 (15)	0.05632 (17)	0.0290 (4)	
C11B	0.9372 (3)	0.67978 (16)	−0.03685 (17)	0.0295 (4)	
H11B	0.9934	0.7112	−0.0997	0.035*	
C12B	0.8477 (3)	0.59132 (16)	−0.03526 (17)	0.0284 (4)	
C13B	0.9012 (3)	0.5872 (2)	−0.21823 (18)	0.0390 (5)	
H13D	0.8889	0.5406	−0.2715	0.058*	
H13E	1.0239	0.5989	−0.2242	0.058*	
H13F	0.8392	0.6530	−0.2265	0.058*	

### Atomic displacement parameters (Å^2^)

**Table:**

	*U*^11^	*U*^22^	*U*^33^	*U*^12^	*U*^13^	*U*^23^
S1A	0.0491 (3)	0.0307 (3)	0.0268 (3)	−0.0122 (2)	−0.0068 (2)	−0.0023 (2)
S1B	0.0523 (3)	0.0293 (3)	0.0280 (3)	−0.0125 (2)	−0.0078 (2)	−0.0018 (2)
O1A	0.0408 (8)	0.0242 (7)	0.0361 (8)	−0.0058 (6)	−0.0108 (7)	−0.0012 (6)
O2A	0.0453 (9)	0.0306 (8)	0.0303 (8)	−0.0094 (7)	−0.0080 (7)	−0.0025 (6)
O3A	0.0459 (9)	0.0298 (8)	0.0283 (8)	−0.0098 (6)	−0.0120 (7)	−0.0016 (6)
O4A	0.0576 (11)	0.0345 (9)	0.0336 (9)	−0.0177 (7)	−0.0058 (8)	0.0015 (7)
O5A	0.0594 (11)	0.0308 (8)	0.0373 (9)	−0.0141 (7)	−0.0190 (8)	−0.0030 (7)
O1B	0.0398 (8)	0.0245 (7)	0.0338 (8)	−0.0050 (6)	−0.0089 (7)	−0.0034 (6)
O2B	0.0456 (9)	0.0313 (8)	0.0304 (8)	−0.0090 (7)	−0.0064 (7)	−0.0042 (6)
O3B	0.0462 (9)	0.0331 (8)	0.0259 (8)	−0.0107 (7)	−0.0078 (7)	−0.0036 (6)
O4B	0.0550 (10)	0.0313 (8)	0.0334 (8)	−0.0120 (7)	−0.0142 (8)	−0.0050 (6)
O5B	0.0488 (10)	0.0351 (9)	0.0348 (9)	−0.0139 (7)	−0.0054 (7)	−0.0014 (7)
N1A	0.0375 (10)	0.0244 (8)	0.0278 (9)	−0.0058 (7)	−0.0070 (7)	−0.0010 (7)
N2A	0.0377 (9)	0.0239 (8)	0.0247 (8)	−0.0047 (7)	−0.0085 (7)	−0.0045 (6)
N3A	0.0342 (9)	0.0237 (8)	0.0337 (10)	−0.0043 (7)	−0.0073 (8)	−0.0035 (7)
N1B	0.0373 (9)	0.0221 (8)	0.0301 (9)	−0.0049 (7)	−0.0060 (7)	−0.0026 (7)
N2B	0.0364 (9)	0.0235 (8)	0.0272 (8)	−0.0034 (7)	−0.0092 (7)	−0.0048 (7)
N3B	0.0345 (9)	0.0266 (9)	0.0330 (9)	−0.0040 (7)	−0.0106 (8)	−0.0018 (7)
C1A	0.0383 (12)	0.0236 (10)	0.0436 (13)	−0.0042 (8)	−0.0129 (10)	−0.0041 (9)
C2A	0.0397 (12)	0.0258 (10)	0.0433 (13)	−0.0013 (9)	−0.0117 (10)	−0.0096 (9)
C3A	0.0372 (11)	0.0333 (11)	0.0339 (11)	−0.0010 (9)	−0.0090 (9)	−0.0089 (9)
C4A	0.0323 (10)	0.0247 (10)	0.0323 (11)	−0.0014 (8)	−0.0072 (9)	−0.0024 (8)
C5A	0.0302 (10)	0.0250 (10)	0.0319 (11)	−0.0010 (8)	−0.0074 (8)	−0.0064 (8)
C6A	0.0319 (10)	0.0238 (9)	0.0301 (10)	−0.0016 (8)	−0.0066 (8)	−0.0045 (8)
C7A	0.0307 (10)	0.0204 (9)	0.0297 (10)	−0.0021 (7)	−0.0067 (8)	−0.0033 (8)
C8A	0.0386 (11)	0.0232 (10)	0.0259 (10)	−0.0022 (8)	−0.0054 (8)	−0.0020 (7)
C9A	0.0389 (11)	0.0252 (10)	0.0281 (10)	−0.0037 (8)	−0.0098 (9)	−0.0038 (8)
C10A	0.0298 (10)	0.0213 (9)	0.0319 (11)	−0.0033 (7)	−0.0064 (8)	−0.0043 (8)
C11A	0.0329 (10)	0.0243 (10)	0.0286 (10)	−0.0031 (8)	−0.0051 (8)	−0.0019 (8)
C12A	0.0310 (10)	0.0253 (10)	0.0268 (10)	−0.0006 (8)	−0.0078 (8)	−0.0050 (8)
C13A	0.0498 (14)	0.0432 (13)	0.0276 (11)	−0.0134 (11)	−0.0119 (10)	0.0004 (9)
C1B	0.0388 (12)	0.0235 (10)	0.0425 (13)	−0.0049 (8)	−0.0135 (10)	−0.0031 (9)
C2B	0.0419 (12)	0.0285 (11)	0.0417 (12)	−0.0011 (9)	−0.0162 (10)	−0.0073 (9)
C3B	0.0431 (12)	0.0306 (11)	0.0348 (11)	0.0012 (9)	−0.0109 (10)	−0.0069 (9)
C4B	0.0347 (11)	0.0234 (10)	0.0333 (11)	0.0005 (8)	−0.0088 (9)	−0.0036 (8)
C5B	0.0343 (11)	0.0249 (10)	0.0313 (10)	0.0003 (8)	−0.0093 (9)	−0.0056 (8)
C6B	0.0320 (10)	0.0224 (9)	0.0298 (10)	−0.0011 (8)	−0.0065 (8)	−0.0039 (8)
C7B	0.0294 (10)	0.0213 (9)	0.0297 (10)	−0.0013 (7)	−0.0076 (8)	−0.0028 (8)
C8B	0.0415 (12)	0.0253 (10)	0.0262 (10)	−0.0035 (8)	−0.0061 (9)	−0.0007 (8)
C9B	0.0418 (12)	0.0270 (10)	0.0266 (10)	−0.0031 (9)	−0.0101 (9)	−0.0038 (8)
C10B	0.0322 (10)	0.0209 (9)	0.0351 (11)	−0.0021 (8)	−0.0096 (9)	−0.0020 (8)
C11B	0.0329 (10)	0.0255 (10)	0.0299 (10)	−0.0038 (8)	−0.0065 (8)	0.0008 (8)
C12B	0.0323 (10)	0.0254 (10)	0.0280 (10)	0.0007 (8)	−0.0075 (8)	−0.0055 (8)
C13B	0.0496 (14)	0.0418 (13)	0.0260 (11)	−0.0118 (10)	−0.0070 (10)	−0.0017 (9)

### Geometric parameters (Å, º)

**Table:**

S1A—C6A	1.668 (2)	C3A—C4A	1.353 (3)
S1B—C6B	1.665 (2)	C3A—H3AA	0.9500
O1A—C1A	1.358 (3)	C4A—C5A	1.462 (3)
O1A—C4A	1.375 (3)	C7A—C8A	1.393 (3)
O2A—C5A	1.226 (3)	C7A—C12A	1.417 (3)
O3A—C12A	1.356 (3)	C8A—C9A	1.385 (3)
O3A—C13A	1.432 (3)	C8A—H8AA	0.9500
O4A—N3A	1.231 (3)	C9A—C10A	1.382 (3)
O5A—N3A	1.229 (3)	C9A—H9AA	0.9500
O1B—C1B	1.363 (3)	C10A—C11A	1.390 (3)
O1B—C4B	1.373 (3)	C11A—C12A	1.385 (3)
O2B—C5B	1.224 (3)	C11A—H11A	0.9500
O3B—C12B	1.357 (3)	C13A—H13A	0.9800
O3B—C13B	1.435 (3)	C13A—H13B	0.9800
O4B—N3B	1.233 (3)	C13A—H13C	0.9800
O5B—N3B	1.227 (3)	C1B—C2B	1.341 (4)
N1A—C5A	1.390 (3)	C1B—H1BB	0.9500
N1A—C6A	1.391 (3)	C2B—C3B	1.421 (3)
N1A—H1AA	0.8800	C2B—H2BB	0.9500
N2A—C6A	1.349 (3)	C3B—C4B	1.356 (3)
N2A—C7A	1.405 (3)	C3B—H3BA	0.9500
N2A—H2AA	0.8800	C4B—C5B	1.456 (3)
N3A—C10A	1.466 (3)	C7B—C8B	1.397 (3)
N1B—C5B	1.389 (3)	C7B—C12B	1.415 (3)
N1B—C6B	1.397 (3)	C8B—C9B	1.383 (3)
N1B—H1BA	0.8800	C8B—H8BA	0.9500
N2B—C6B	1.348 (3)	C9B—C10B	1.380 (3)
N2B—C7B	1.406 (3)	C9B—H9BA	0.9500
N2B—H2BA	0.8800	C10B—C11B	1.393 (3)
N3B—C10B	1.464 (3)	C11B—C12B	1.382 (3)
C1A—C2A	1.349 (4)	C11B—H11B	0.9500
C1A—H1AB	0.9500	C13B—H13D	0.9800
C2A—C3A	1.418 (3)	C13B—H13E	0.9800
C2A—H2AB	0.9500	C13B—H13F	0.9800
			
C1A—O1A—C4A	106.15 (18)	C12A—C11A—H11A	121.0
C12A—O3A—C13A	118.46 (17)	C10A—C11A—H11A	121.0
C1B—O1B—C4B	106.16 (18)	O3A—C12A—C11A	124.7 (2)
C12B—O3B—C13B	118.39 (18)	O3A—C12A—C7A	114.87 (18)
C5A—N1A—C6A	128.54 (19)	C11A—C12A—C7A	120.42 (19)
C5A—N1A—H1AA	115.7	O3A—C13A—H13A	109.5
C6A—N1A—H1AA	115.7	O3A—C13A—H13B	109.5
C6A—N2A—C7A	130.85 (19)	H13A—C13A—H13B	109.5
C6A—N2A—H2AA	114.6	O3A—C13A—H13C	109.5
C7A—N2A—H2AA	114.6	H13A—C13A—H13C	109.5
O5A—N3A—O4A	123.17 (19)	H13B—C13A—H13C	109.5
O5A—N3A—C10A	118.71 (19)	C2B—C1B—O1B	110.3 (2)
O4A—N3A—C10A	118.12 (18)	C2B—C1B—H1BB	124.8
C5B—N1B—C6B	128.25 (19)	O1B—C1B—H1BB	124.8
C5B—N1B—H1BA	115.9	C1B—C2B—C3B	107.5 (2)
C6B—N1B—H1BA	115.9	C1B—C2B—H2BB	126.3
C6B—N2B—C7B	130.55 (19)	C3B—C2B—H2BB	126.3
C6B—N2B—H2BA	114.7	C4B—C3B—C2B	105.6 (2)
C7B—N2B—H2BA	114.7	C4B—C3B—H3BA	127.2
O5B—N3B—O4B	123.18 (19)	C2B—C3B—H3BA	127.2
O5B—N3B—C10B	118.14 (19)	C3B—C4B—O1B	110.5 (2)
O4B—N3B—C10B	118.68 (19)	C3B—C4B—C5B	131.3 (2)
C2A—C1A—O1A	110.5 (2)	O1B—C4B—C5B	118.24 (19)
C2A—C1A—H1AB	124.7	O2B—C5B—N1B	123.56 (19)
O1A—C1A—H1AB	124.7	O2B—C5B—C4B	122.1 (2)
C1A—C2A—C3A	106.9 (2)	N1B—C5B—C4B	114.33 (19)
C1A—C2A—H2AB	126.5	N2B—C6B—N1B	114.56 (19)
C3A—C2A—H2AB	126.5	N2B—C6B—S1B	127.98 (16)
C4A—C3A—C2A	106.0 (2)	N1B—C6B—S1B	117.46 (16)
C4A—C3A—H3AA	127.0	C8B—C7B—N2B	126.6 (2)
C2A—C3A—H3AA	127.0	C8B—C7B—C12B	119.26 (19)
C3A—C4A—O1A	110.37 (19)	N2B—C7B—C12B	114.10 (19)
C3A—C4A—C5A	131.5 (2)	C9B—C8B—C7B	120.6 (2)
O1A—C4A—C5A	118.06 (19)	C9B—C8B—H8BA	119.7
O2A—C5A—N1A	123.84 (19)	C7B—C8B—H8BA	119.7
O2A—C5A—C4A	121.8 (2)	C10B—C9B—C8B	118.8 (2)
N1A—C5A—C4A	114.39 (19)	C10B—C9B—H9BA	120.6
N2A—C6A—N1A	114.52 (19)	C8B—C9B—H9BA	120.6
N2A—C6A—S1A	127.94 (16)	C9B—C10B—C11B	122.7 (2)
N1A—C6A—S1A	117.54 (16)	C9B—C10B—N3B	119.4 (2)
C8A—C7A—N2A	126.9 (2)	C11B—C10B—N3B	117.8 (2)
C8A—C7A—C12A	119.44 (19)	C12B—C11B—C10B	118.2 (2)
N2A—C7A—C12A	113.62 (18)	C12B—C11B—H11B	120.9
C9A—C8A—C7A	120.5 (2)	C10B—C11B—H11B	120.9
C9A—C8A—H8AA	119.7	O3B—C12B—C11B	125.0 (2)
C7A—C8A—H8AA	119.7	O3B—C12B—C7B	114.55 (18)
C10A—C9A—C8A	118.63 (19)	C11B—C12B—C7B	120.5 (2)
C10A—C9A—H9AA	120.7	O3B—C13B—H13D	109.5
C8A—C9A—H9AA	120.7	O3B—C13B—H13E	109.5
C9A—C10A—C11A	123.01 (19)	H13D—C13B—H13E	109.5
C9A—C10A—N3A	118.99 (19)	O3B—C13B—H13F	109.5
C11A—C10A—N3A	118.00 (19)	H13D—C13B—H13F	109.5
C12A—C11A—C10A	118.0 (2)	H13E—C13B—H13F	109.5
			
C4A—O1A—C1A—C2A	−0.4 (3)	C4B—O1B—C1B—C2B	0.3 (3)
O1A—C1A—C2A—C3A	0.4 (3)	O1B—C1B—C2B—C3B	0.2 (3)
C1A—C2A—C3A—C4A	−0.3 (3)	C1B—C2B—C3B—C4B	−0.5 (3)
C2A—C3A—C4A—O1A	0.0 (3)	C2B—C3B—C4B—O1B	0.7 (3)
C2A—C3A—C4A—C5A	178.1 (2)	C2B—C3B—C4B—C5B	−179.2 (2)
C1A—O1A—C4A—C3A	0.2 (3)	C1B—O1B—C4B—C3B	−0.6 (2)
C1A—O1A—C4A—C5A	−178.12 (19)	C1B—O1B—C4B—C5B	179.31 (19)
C6A—N1A—C5A—O2A	−2.6 (4)	C6B—N1B—C5B—O2B	−0.4 (4)
C6A—N1A—C5A—C4A	177.3 (2)	C6B—N1B—C5B—C4B	179.5 (2)
C3A—C4A—C5A—O2A	−1.4 (4)	C3B—C4B—C5B—O2B	6.9 (4)
O1A—C4A—C5A—O2A	176.6 (2)	O1B—C4B—C5B—O2B	−173.0 (2)
C3A—C4A—C5A—N1A	178.8 (2)	C3B—C4B—C5B—N1B	−173.1 (2)
O1A—C4A—C5A—N1A	−3.2 (3)	O1B—C4B—C5B—N1B	7.0 (3)
C7A—N2A—C6A—N1A	178.6 (2)	C7B—N2B—C6B—N1B	−179.1 (2)
C7A—N2A—C6A—S1A	−0.9 (4)	C7B—N2B—C6B—S1B	0.4 (4)
C5A—N1A—C6A—N2A	1.7 (3)	C5B—N1B—C6B—N2B	8.1 (3)
C5A—N1A—C6A—S1A	−178.79 (18)	C5B—N1B—C6B—S1B	−171.41 (18)
C6A—N2A—C7A—C8A	−1.7 (4)	C6B—N2B—C7B—C8B	−8.5 (4)
C6A—N2A—C7A—C12A	178.8 (2)	C6B—N2B—C7B—C12B	172.3 (2)
N2A—C7A—C8A—C9A	−179.6 (2)	N2B—C7B—C8B—C9B	−179.9 (2)
C12A—C7A—C8A—C9A	−0.2 (3)	C12B—C7B—C8B—C9B	−0.7 (3)
C7A—C8A—C9A—C10A	−0.1 (3)	C7B—C8B—C9B—C10B	−0.8 (3)
C8A—C9A—C10A—C11A	0.2 (3)	C8B—C9B—C10B—C11B	1.5 (4)
C8A—C9A—C10A—N3A	−179.6 (2)	C8B—C9B—C10B—N3B	−178.69 (19)
O5A—N3A—C10A—C9A	1.8 (3)	O5B—N3B—C10B—C9B	175.9 (2)
O4A—N3A—C10A—C9A	−178.1 (2)	O4B—N3B—C10B—C9B	−4.1 (3)
O5A—N3A—C10A—C11A	−178.0 (2)	O5B—N3B—C10B—C11B	−4.3 (3)
O4A—N3A—C10A—C11A	2.1 (3)	O4B—N3B—C10B—C11B	175.7 (2)
C9A—C10A—C11A—C12A	0.1 (3)	C9B—C10B—C11B—C12B	−0.6 (3)
N3A—C10A—C11A—C12A	179.86 (19)	N3B—C10B—C11B—C12B	179.55 (19)
C13A—O3A—C12A—C11A	−4.4 (3)	C13B—O3B—C12B—C11B	−3.6 (3)
C13A—O3A—C12A—C7A	175.4 (2)	C13B—O3B—C12B—C7B	176.9 (2)
C10A—C11A—C12A—O3A	179.4 (2)	C10B—C11B—C12B—O3B	179.6 (2)
C10A—C11A—C12A—C7A	−0.3 (3)	C10B—C11B—C12B—C7B	−0.9 (3)
C8A—C7A—C12A—O3A	−179.34 (19)	C8B—C7B—C12B—O3B	−178.96 (19)
N2A—C7A—C12A—O3A	0.1 (3)	N2B—C7B—C12B—O3B	0.4 (3)
C8A—C7A—C12A—C11A	0.4 (3)	C8B—C7B—C12B—C11B	1.5 (3)
N2A—C7A—C12A—C11A	179.88 (19)	N2B—C7B—C12B—C11B	−179.13 (19)

### Hydrogen-bond geometry (Å, º)

**Table:**

*D*—H···*A*	*D*—H	H···*A*	*D*···*A*	*D*—H···*A*
N1*A*—H1*AA*···O1*A*	0.88	2.24	2.684 (2)	111
N2*A*—H2*AA*···O2*A*	0.88	1.91	2.654 (2)	142
N2*A*—H2*AA*···O3*A*	0.88	2.09	2.552 (2)	112
N1*B*—H1*BA*···O1*B*	0.88	2.25	2.683 (2)	111
N2*B*—H2*BA*···O2*B*	0.88	1.92	2.653 (2)	140
N2*B*—H2*BA*···O3*B*	0.88	2.11	2.554 (2)	111
C8*A*—H8*AA*···S1*A*	0.95	2.52	3.198 (2)	129
C8*B*—H8*BA*···S1*B*	0.95	2.52	3.189 (2)	128
